# A Simulation Study of a Radiofrequency Localization System for Tracking Patient Motion in Radiotherapy

**DOI:** 10.3390/s16040534

**Published:** 2016-04-13

**Authors:** Mark Ostyn, Siyong Kim, Woon-Hong Yeo

**Affiliations:** 1 Radiation Oncology, Medical Physics Graduate Program, School of Medicine, Virginia Commonwealth University, Richmond, VA 23298, USA; ostynmr@vcu.edu (M.O.); siyong.kim@vcuhealth.org (S.K.); 2 Department of Mechanical and Nuclear Engineering, School of Engineering, Virginia Commonwealth University, Richmond, VA 23284, USA; 3 Center for Rehabilitation Science and Engineering, School of Medicine, Virginia Commonwealth University, Richmond, VA 23298, USA

**Keywords:** radiotherapy, localization, direction of arrival, angulation, Monte Carlo simulation, intrafraction motion

## Abstract

One of the most widely used tools in cancer treatment is external beam radiotherapy. However, the major risk involved in radiotherapy is excess radiation dose to healthy tissue, exacerbated by patient motion. Here, we present a simulation study of a potential radiofrequency (RF) localization system designed to track intrafraction motion (target motion during the radiation treatment). This system includes skin-wearable RF beacons and an external tracking system. We develop an analytical model for direction of arrival measurement with radio frequencies (GHz range) for use in a localization estimate. We use a Monte Carlo simulation to investigate the relationship between a localization estimate and angular resolution of sensors (signal receivers) in a simulated room. The results indicate that the external sensor needs an angular resolution of about 0.03 degrees to achieve millimeter-level localization accuracy in a treatment room. This fundamental study of a novel RF localization system offers the groundwork to design a radiotherapy-compatible patient positioning system for active motion compensation.

## 1. Introduction

**Figure 1:**
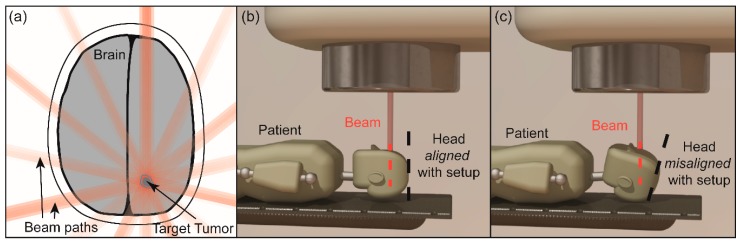
(**a**) Example of conformal radiotherapy for targeting a brain tumor; (**b**,**c**) Schematic illustrations for a patient receiving external beam radiation therapy for brain cancer. Initially, the beam is delivered to the right target with a good alignment (**b**), but the motion causes the head misalignment (**c**), which makes an error relative to the planned delivery.

External beam radiation therapy is one of the most significant tools available in modern cancer treatment. Linear accelerators generate high-energy photons and careful planning directs the output into patient tumors. These X-rays introduce an ionizing dose of radiation into matter along the path of the beam. The received dose damages DNA at the molecular level, which increases the likelihood of cell death. Healthy tissue along the beam path is spared from receiving excessive dose by spreading dose delivery over several beam angles (Figure 1a). Most treatments deliver the cumulative dose in a series of 15–30 min fractions over the course of 2–8 weeks, which grants healthy tissue sufficient time for DNA repair.

New delivery techniques, such as intensity-modulated radiation therapy or volume-modulated arc therapy, allow precise delivery of the dose to a set volume in space, conforming to the shape of the tumor and sparing healthy tissue. These types of treatments are often characterized by steep dose gradients between the target region and normal tissue, which necessitates accurate positioning during treatment. Precise information about the position, orientation, and motion of the tumor is often unknown during dose delivery. Thus, oncologists prescribe a volumetric margin of necessary size to deliver the prescribed dose to the disease site volume, for a given level of spatial uncertainty [1]. Larger margins increase the amount of healthy tissue receiving cell-lethal dose and may overlap with critical organs such as the brain, lungs, heart, or bowels. Thus, modern treatment planning requires sufficiently large margins to ensure tumor coverage as well as necessarily small margins to avoid delivering excess dose to healthy tissue. The magnitude of the spatial uncertainties must be well known and minimized.

The most notable sources of uncertainty are improper alignment of a patient with the prescribed field. An example in Figure 1b,c shows intrafraction motion, caused by patient movement during the treatment. Setup errors have been minimized by employment of immobilization equipment and alignment of fiducial markers with static laser grids, but intrafraction motion has only recently been studied. Optical systems, such as surface tracking [2,3,4,5,6] or marker tracking [7], have been used successfully to monitor intrafraction motion in several disease sites. However, these systems are not robust against changes in patient size and shape and require line-of-sight alignment, which may become obstructed by the linear accelerator gantry. Radiographic systems that overcome many of these challenges exist [8], but they issue further unnecessary radiation dose when used, especially if used in real-time. Calypso Medical Technologies (now owned by Varian Medical Systems) developed a radiofrequency (RF) tracking system that utilizes transponders implanted in tumors to track the interior disease in real-time [8,9]. Although this system successfully meets this real-rime tracking goal and overcomes many of the disadvantages inherent to optical systems, the bulky RF array is often cumbersome to use in the clinic and prevents the use of radiographic imaging while in use, which definitively gives the position of interior anatomy rather than Calypso’s surrogate positions. Further, Calypso is constrained to extracranial sites, limiting its full clinical utility. Lastly, while few complications have occurred involving the surgical implant of seeds, the necessity of surgical procedures is undesirable.

In this paper, we introduce a novel RF tracking system capable of monitoring the patient motion in near real-time, which can overcome all of the aforementioned disadvantages. Such a system would consist of series of skin-wearable transmitters [10,11,12] gently affixed to a patient and an external sensor network that tracks the patient position and attitude in real-time via estimate of the transmitters’ positions. RF waves have intrinsic advantages over other photonic means because radio waves readily propagate through many materials and do not deliver any radiation dose. In addition, such transmitters placed at strategic anatomical sites outside of the radiation field would be resilient against the issue of skin deformation, normally seen by optical systems; identification of each transmitter on a patient facilitates tracking multiple rigid bodies.

## 2. Materials and Methods

### 2.1. Localization System

**Figure 2:**
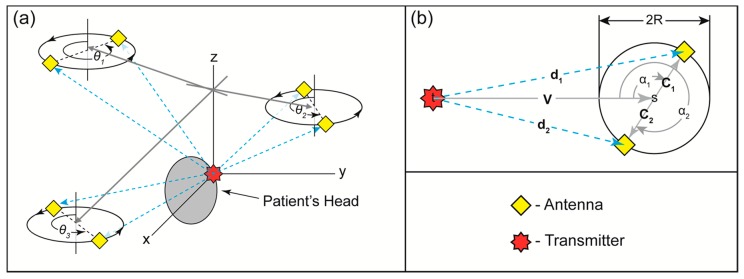
(**a**) A network of sensors with antennas used in parallel to find the location of the transmitter in a patient’s head. The transmitter sends a radiofrequency (RF) signal to the antennas as they turn through angle *θ*, which is measured individually by each antenna in each sensor. The gray arrows represent the estimated direction of arrival by each sensor projected into their planes of rotation; (**b**) Geometric abstract of a single sensor having two antennas used to find the direction vector *V* of the transmitter. *C_i_*, *d_i_*, and *α_i_* refer to the distance from the center of the sensor to the *i*-th antenna, the distance from the transmitter to the *i*-th antenna, and the angular position of *i*-th antenna, respectively. Location *t* refers to the geometric location of the transmitter and location *s* refers to the location of the center of the sensor. The diameter of the entire sensor is 2R. The intersection of the direction vector *V* from each sensor finds the location of the transmitter.

We designed a localization system that uses the direction of arrival measurements in an RF sensor network to angulate skin-wearable transmitters on a patient. This technique allows a precise localization of transmitters, mounted on a patient, without demanding strict timing such as global positioning system [13]. Many current approaches [14,15,16] measure the direction of arrival of an RF signal by comparing the phase difference of an RF wave between elements in static antenna arrays. We propose a similar system, but with two major variations: a rotating array platform and operating at two significantly different frequencies. This sensor system can find the direction of patient-mounted transmitters in a 2D plane with high accuracy (~0.1°). A generic prototype of this sensor design was simulated by a programming language (MATLAB, MathWorks, Natick, MA, USA). A network of sensors then used the combined direction measurements to find the location of the transmitters in a 2D plane (Figure 2a). A closed form solution based on real measurements is extremely unlikely to exist for any positioning system, so least squares estimation of the position is required. In order to estimate the accuracy of the least squares solution, we used a Monte Carlo simulation. The simulation estimates the magnitude of the positioning error based on the number of sensors in the network and the angular resolution of the sensors.

### 2.2. Direction of Arrival Measurements

**Figure 3:**
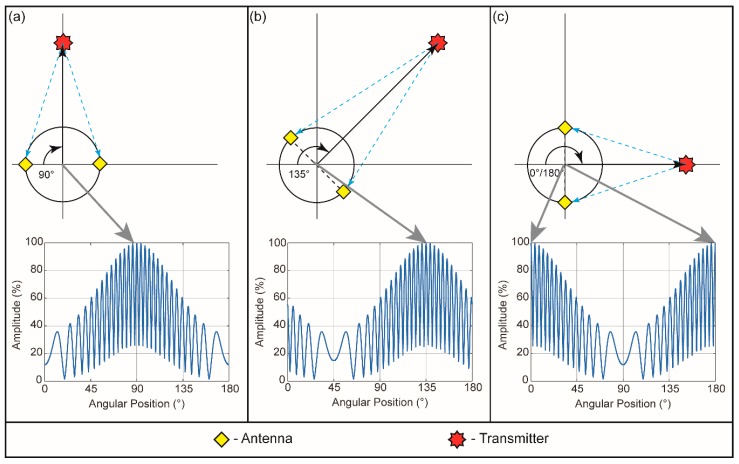
Spatial geometry of a single sensor with two antennas at the selected locations and the sensor’s signal response for one-half revolution (*not to scale*). The central peak of the signal shape represents the direction used for the narrow search. The three selected transmitter positions shown are: (**a**) 90° with respect to the coordinate system; (**b**) 135° with respect to the coordinate system; and (**c**) 0°/180° with respect to the coordinate system. In all cases, the distance between the center of the sensor and the transmitter was 2 m in the horizontal plane and 1 meter in the vertical plane.

Figure 2b illustrates the geometry of the external sensor system. The system consists of two antennas placed at opposite sides of a circle (diameter: 2R) which rotates around its central axis. The relation between the signals received by these antennas determines the direction vector toward the patient-mounted transmitter projected onto the 2D plane of rotation. Each transmitter on a patient emits a sine wave, *x*(*t*), for a given duration, which consists of two frequencies (1.5 and 48 GHz) as a compromise between antenna spatial constraints and available spectra. As the sensor system rotates, the signals from two antennas are multiplied together and the resultant amplitude is measured. In the simulation, the transmitter was placed at origin and the center of the sensor was placed at two meters away in the horizontal plane and one meter above the plane of the transmitter. In a 2D plane, this corresponds to the setup described in Figure 3. Because the lower frequency component has a wavelength equal to twice the length of the sensor’s diameter, the resultant interfered signal oscillates at the rate of the sensor rotation, and the angular position of the peak corresponds to the direction of arrival. The high frequency component creates a secondary oscillation with a predictable pattern that facilitates identification of the center of the peak (Figure 3). In a real setting in the treatment room, one-half of a sensor rotation could be used as a coarse measurement to find the direction to the nearest 5°, and then a high-resolution scan may be performed over a much smaller arc to find the direction at the limitations of the method (~0.05°). This method can accurately determine the 2D projected direction of arrival vector even in cases where the out-of-plane angle is severe (greater than 80°). Because the wavelength of the high frequency signal is very short (6.6 mm) the differences in path length between the two antennas is still distinguishable. In our simulation, the following equation creates a time-dynamic transmitted wave:

(1) $$x\left(t\right)=\;A_{1}\sin\left(2\pi f_{1}t+\;\varphi_{1}\right)+\;A_{2}\sin\left(2\pi f_{2}t+\;\varphi_{2}\right)$$

where *A* is the amplitude of each of the two waves, *f* is the frequency of each wave, *t* is the sampled point in time, and *ϕ* is the initial phase of each wave. The sine wave is sampled at 150 THz over a 33 ns pulse. This corresponds to 50 wavelengths of the lower frequency and approximately 1500 wavelengths of the higher frequency over 2.5 × 10^5^ points, or 5000 points per wavelength of the low frequency and approximately 170 points per wavelength of the high frequency. This sampling rate was chosen as compromise between fully modeling the complete sine wave of the high frequency and conserving system memory. The starting phases of the wave components were chosen from a uniform random distribution between 0 and 2π. The amplitudes of both waves are assumed equal in strength. The wavelength of the lower frequency component is calculated according to the speed of light in air, and is termed as the carrier wavelength.

Vector analysis defines the location of the transmitter as point ***t*** in 3D space. The center of a given sensor is defined as point ***s***, and the angular position of the *i*-th antenna in the plane of the sensor’s rotation is defined as *α*_i_. The vector ***V*** between the transmitter’s location and the center of the sensor is defined as:

(2) $$\hat{V}=\;s-t$$

The vectors from the center of the sensor to the *i*-th antenna receiver was then calculated for angular positions *j*:

(3) $${\hat{C}}_{i,j}=R\times \left[\cos\alpha_{i,j},\sin\alpha_{i,j},0\right]$$

The distance between the transmitter and each *i*-th receiver at every *j*-th sampled angular position was then found as the magnitude of the vector connecting between the transmitter and each receiver:

(4) $$d_{i,j}=║\hat{V}+{\hat{C}}_{i,j}║$$

This distance is then converted to fractions of the carrier wavelength, *m_i_*_,*j*_. At each angular position *j*, each receiver reads the transmitted signal after the respective number of carrier wavelengths of the transmitted signal, *m_i_*_,*j*_ + *M*, for *n* wavelengths, where *M* represents the minimum number of carrier wavelengths to wait before the signal is read. This “wait” parameter was included to ensure the antennas did not attempt to read the signal before the first index in the transmitted signal. We used *M* = 5 and *n* = 10 over 2000 uniformly spaced angular positions between 0° and 180° in the simulation. In reality, the device will very likely rotate slower than the 37.5 kHz that this math suggests (10 periods of a 1.5 GHz wave per angle for 2000 angles per half revolution). We have chosen *n* = 10 wavelengths interfered at each angle as a compromise between obtaining sufficient signal to read the amplitude and data storage. In a practical implementation, this will be achieved through analog means.

This process simulates the phase difference *φ*, measured between the two receiving antennas at the same instant. Uniform Gaussian white noise is added at a given signal-to-noise ratio (SNR). Real clinical settings contain numerous potential RF reflectors, which could cause a very noisy RF environment. Therefore, we used an SNR value of 2 as a pessimistic assumption. The signals were then multiplied and the resultant amplitude was recorded against each sensor angle αj. A median filter was used to smooth the resulting data. The “findpeaks” algorithm of MATLAB’s Signal Processing Toolbox [17] was then used to identify the locations of the signal peaks by taking the location of the local maxima as the location of the peak. This process was repeated over 5000 iterations in a 4° search space of the simulated transmitter’s known position to judge the accuracy of the direction finding algorithm.

### 2.3. Angulation Uncertainty Estimation

**Figure 4:**
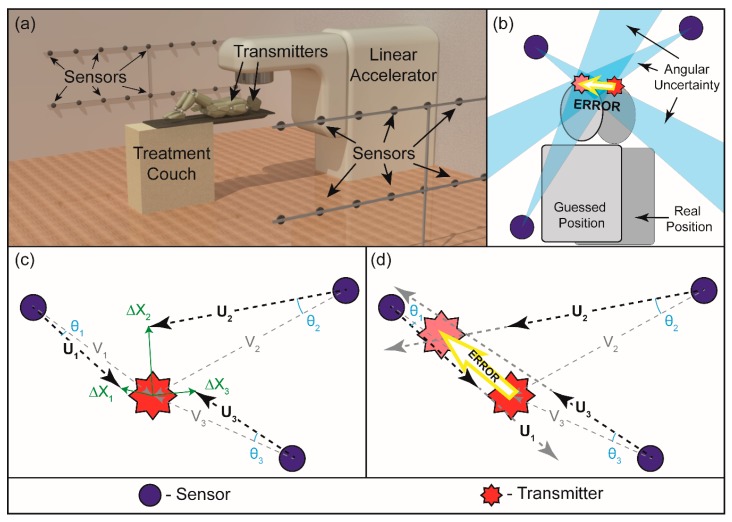
(**a**) Three-dimension rendering of sensor grid in the clinical setting. Transmitters are attached to the patient; (**b**) Rationale for creating the Monte Carlo simulation: angular uncertainty exists in direction measurements, which can lead to errors in the localization process; (**c**) Process of the Monte Carlo simulation: angular uncertainty *θ* is created by adding a vector Δ*X* to the true vector between sensor and transmitter *V*, resulting in a simulated mis-measurement of direction *U*. The vectors Δ*X_i_* are randomly selected from a 3D Gaussian distribution; (**d**) Continued process of the Monte Carlo simulation: a least squares calculation is used to find the best-fit intersection of the mis-measured direction vectors, *U_i_*, and the error is taken as the difference between the intersections of the mis-measured vectors *U_i_* and the intersection of the true vectors *V_i_*. The error is compared to the average angular uncertainty *θ_i_*.

Multiple sensors are required to localize each transmitter’s signal in 3D space. Figure 4a displays the general layout of a practical scenario including transmitters on a patient and an array of sensors surrounded in a treatment room. In our simulation, the external sensor locations were randomly distributed in a room-sized hemispherical shell, centered at the simulated transmitter, to investigate the relationship between the number of direction measurements and localization estimate. The polar and azimuthal coordinates of each sensor were selected in a uniform 2π space. The radial distance from the transmitter to each receiver was taken from a uniform distribution between 3.5 and 4.5 m; the approximate size of a treatment room. The relationship between the angular resolution of the sensors and the accuracy of the position estimate was investigated by performing multiple iterations of estimation based on the same physical setup but varying the magnitude of the uncertainty in the angular measurement (Figure 4b).

We also investigated the relationship between the number of sensors and the physical arrangement of them. The initial 3D direction vectors between each sensor and transmitter was calculated as:

(5) $${\hat{V}}_{i}=\frac{s_{i}-p}{∥s_{i}-p∥}$$

where the position ***s_i_*** represents the position of the *i*-th sensor and ***p*** represents the position of the transmitter. In order to simulate uncertainty in the direction of arrival measurement spatial variation, **∆*X_i_*_,*j*_** was added to the transmitter’s position for direction of arrival measurement where **∆*X_i_*_,*j*_** is taken from a 3D Gaussian distribution of standard deviation σ*_j_* (Figure 4c). Values of σ*_j_* were varied exponentially between 1.5^−28^ and 1.5^−2^ m (0.012–440 mm) to observe a wide range of angular resolutions.

(6) $${\hat{U}}_{i,j}=\frac{s_{i}-\left(p+\Delta {\hat{X}}_{i,j}\left(\sigma_{j}\right)\right)}{∥s_{i}-\left(p+\Delta {\hat{X}}_{i,j}\left(\sigma_{j}\right)\right)∥}$$

A least squares estimate of the position, ***t_j_***, was calculated based on these new direction vectors for each magnitude of angular resolution σ*_j_*.

(7) $$t_{j}=\;{\left(\underset{i}{\sum }\left(I-{\hat{U}}_{i,j}{\hat{U}}_{i,j}{}^{T}\right)\right)}^{-1}\left(\underset{i}{\sum }\left(I-{\hat{U}}_{i,j}{\hat{U}}_{i,j}{}^{T}\right)s_{i}\right)$$

where ***I*** represents a 3 × 3 identity matrix. The magnitude of the error in the estimated position was then found for each level of σ*_j_*.

(8) $$Error_{j}=∥t_{j}-p∥$$

For each level of σ*_j_*, the average angular miss was calculated by taking the average of the dot product between the true direction vectors from the transmitter and sensors and the simulated miss.

(9) $$\bar{\theta_{j}}=\;\bar{\cos^{-1}\left({\hat{U}}_{i,j}\cdot {\hat{V}}_{i}\right)}$$

The mean angular miss *θ* serves as a surrogate for angular uncertainty and was compared to the error in estimated position for each σ*_j_* (Figure 4d). This process had 5000 iterations for each σ*_j_* to obtain statistically acceptable ranges of error for a given angular uncertainty. The number of sensors in total varied by 5, 50, and 500 in the network. This calculation was performed to observe the effect of increasing the number of vectors available for angulation analysis.

## 3. Results

### 3.1. Direction of Arrival Measurements

**Figure 5:**
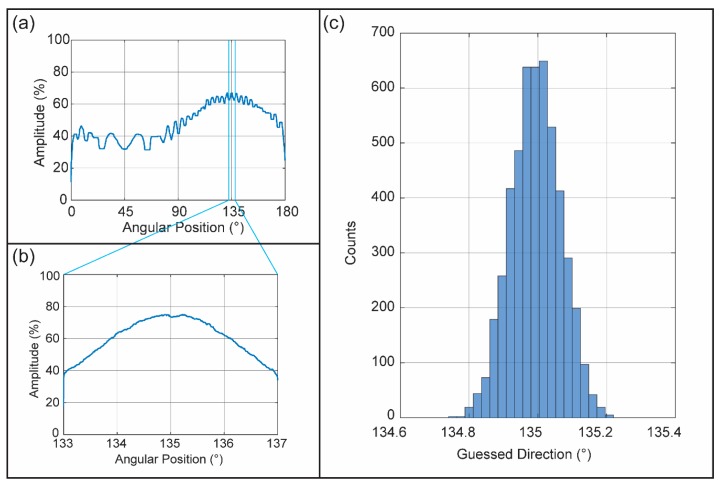
(**a**) Sensor signal response for one-half rotation when the transmitter was located at 135° with respect to the coordinate system with signal-to-noise ratio (SNR) = 2; (**b**) Signal response for the 4° high resolution scan area when the transmitter was at 135° with respect to the coordinate system with SNR = 2; (**c**) Histogram of the results of the direction-finding program used on the high-resolution scan area in (**b**). Over 5000 iterations, the program found the direction of the transmitter to be 134.99° ± 0.07° compared to a ground-truth value of 135°.

Figure 5a shows the simulated results of the direction of signal arrival. The measured data demonstrates that the product of signals from two rotating antennas in the external system forms a signal that may be easily identified [17]. The signal rises from a minimal value at 45° rotation (when the antennas are in line with the signal path) to a maximum value at 135° rotation (when the vector between the antennas is perpendicular to the signal direction). The pattern repeats every 180° because the sensor has 2-fold rotational symmetry. The interference between the mixed-frequency signals (1.5 + 48 GHz) creates a pattern as the antenna array platform rotates, and the ratio of the two frequencies determines the number of signal peaks between 0° and 180° (Figure 5a,b). The central peak of the mixed-frequency signal corresponds to the direction of signal arrival, which is located in angular space by the signal analysis. A peak-finding algorithm found the peak of the high-resolution signal; based on 5000 iterations of this process, the program found the direction of the transmitter to be 134.99° ± 0.07° compared to a ground-truth value of 135° (Figure 5c).

### 3.2. Angulation Uncertainty Analysis

**Figure 6:**
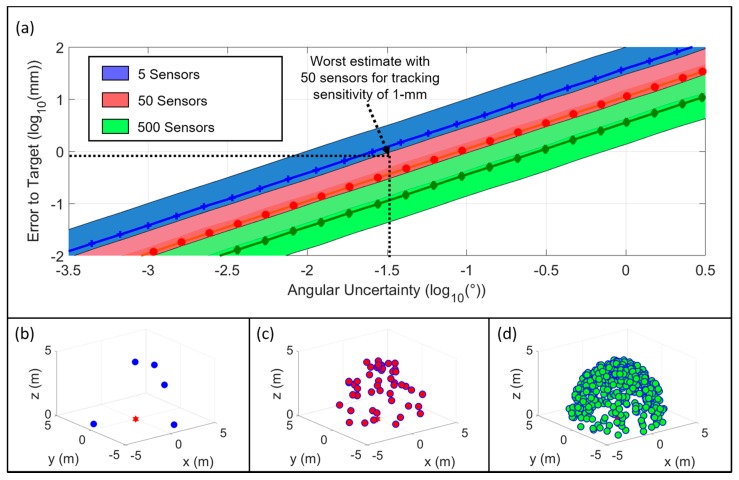
(**a**) Relationship between angular precision and error in least squares estimation of the transmitter position. Filled area represents two standard deviations of Monte Carlo simulation results. Better results are closer to the bottom of the graph, therefore worst-case scenarios for a given number of sensors is upper bound for each filled area; (**b**–**d**) Spatial distribution of sensors for the 5-sensor case (**b**), 50-sensor case (**c**), and 500-sensor case (**d**), respectively.

The relationship between the direction of signal arrival measurement angular uncertainty and the mean error of the position estimate presents the linear patterns (Figure 6a). The result shows that increased number of sensors (Figure 6b–d) improves the position estimation for a given angular resolution, but follows a logarithmic rate of improvement. The logarithmic plot in Figure 6a shows that the slopes of lines are parallel, which means that the rate of estimation improvement with respect to angular resolution is proportional to the number of direction estimates. Our system aims to achieve tracking sensitivity less than 1 mm, which is about the same scale as the typical dose margins in conformal radiotherapy. Therefore, the direction of arrival sensors require an angular resolution near 10^−1.5^ degrees (~0.03°) to achieve the ideal accuracy, which is based on the assumption that the final design will include 50 sensors. The criterion of consistency is based on maintaining the 1-mm sensitivity with 50 sensors in 96% of all estimates. Given that this estimate is an order of magnitude estimate, we conclude that our simulated sensor accuracy and precision is sufficient to track the position of a transmitter on a patient.

## 4. Discussion and Conlusions

In a clinical environment, RF-based tracking equipment is largely untested. Potential RF reflectors include radiotherapy linear accelerators and simulation computed tomography scanners. In this work, we have introduced a RF localization system for accurate tracking of patient motion in radiotherapy, which would provide high accuracy and robustness even in situations where the true signal may be difficult to distinguish from the noise using RF waves. Our system also calls for the use of components that operate in the K-band of the electromagnetic spectrum, which are not widely available in normal commercial channels and less investigated in scientific and engineering literature. RF waves in this spectral range may have different propagation characteristics, which could introduce multi-path noise. The use of these components theoretically provides a significant improvement to our measurements, which justifies their use in tracking patient motion.

We have used 2000 discrete angular points per half rotation for simulating the direction of arrival sensor. In a practical setting, we foresee a system where the sensor would continuously rotate at a constant speed (~10 Hz) while the signals from both antennas are interfered by analog means and the resulting signal is measured every 0.1°, for a total of 1800 data points per half rotation. This calls for a 36 kHz sampling rate, which we believe is feasible. Several transmitters could be used together by serial polling, though maintaining 10 Hz resolution necessary for measuring motion from breathing would require faster sampling rates.

We observed that two parameters can reduce the error in the positioning estimation: improving the angular resolution and increasing the number of sensors. In our simulation of the angulation process, we chose to model the sensors in a uniformly distributed random pattern. This was done to solve for the generic case, because the physical arrangement of sensors was found to have very little impact on the result of the angulation, as long as the transmitter was not in a position past 85° above or below the plane of rotation. In a real setting, the sensors may be positioned in whatever manner is convenient for each individual clinic. Due to practicality reasons, the total number of sensors is constrained to be less than 100, and the receiver-transmitter distance is constrained by the treatment room geometry. Therefore, we have focused a great amount of effort into methods that can improve the angular resolution of our direction of arrival analysis software. Based on standard room sizes and assumption that about 50 sensors will be used, we estimate that an angular resolution near 0.03° is necessary to achieve position estimate errors less than 1 mm. We are optimistic that achieving the resolution of 0.01° is possible with better peak detection algorithms, even in poor SNR environments, which could lead to 1-mm accuracy while using fewer sensors.

In a clinic, the metal gantry of a linear accelerator may move between some of the sensors and the transmitters, which can cause reflections of the RF signal and cause a misreport of the direction of arrival at those obstructed sensors. Since the position of the gantry is well-known at all times, the direction estimate from potentially obstructed sensors could be ignored for localization purposes. This is a potential strength over optical systems, which cannot ignore the input of a single camera if obstructed by the gantry and still provide useful localization information.

Our proposed system has some limitations. Unlike radiographic techniques and the Calypso system, our technique only provides localization information about discrete points on the patient’s surface as opposed to information about the interior anatomy. Therefore, we suggest that our technique be used in conjunction with radiographic techniques for fraction-to-fraction setup to ensure that interior anatomy is well-aligned. Our system is designed to provide reliable intrafraction motion tracking as conveniently and comfortably as possible. We believe that the combination of the ability to freely choose localization points and the ability to track multiple rigid bodies provides a significant advantage over competing optical methods.

The skin wearable transmitters also require further investigation. Patients will need to wear the transmitters over the course of the treatment, which may take several weeks. A few skin-wearable devices [12,18] have been demonstrated to maintain functionality while worn for up to two weeks, even with exercising, showering, and normal living conditions. Therefore, longer periods of wear may be feasible.

Overall, we have introduced a new RF localization system that overcomes the main weaknesses of existing intrafraction motion tracking systems [5,19,20]. This system is robust against changes in patient anatomy and provides real-time tracking of changes in complex patient positioning, without requiring line-of-sight detection and avoiding extra dose to a patient. The practical implementation requires further investigation; the involvement of many mechanical components in sensors may require frequent maintenance. However, the analog nature of the system grants increased precision in measurement, which justifies the risk of maintenance. Future work will include the design of external sensors to implement this analytical and simulation study and skin-wearable electronics [12,18] mounted on a patient for signal transmission. Additionally, the system will include tracking the transmitter in 3D, likely by combining the measurements from two orthogonal 2D localization systems.
